# Prevalence of Internet Gaming Disorder, Depression, and Anxiety Symptoms Before and After the Pandemic

**DOI:** 10.3390/healthcare12232471

**Published:** 2024-12-06

**Authors:** Kira Bailey, Audrey Propp, Maria Alonso

**Affiliations:** David O. Robbins Neuroscience Program, Psychology Department, Ohio Wesleyan University, Delaware, OH 43015, USA

**Keywords:** anxiety, depression, internet gaming disorder, students, video games

## Abstract

“Internet gaming disorder” (IGD) is a condition for further study in the DSM-5, with its prevalence estimated to be anywhere from 0.7% to 27.5% depending on the methodology used to measure it. Previous research has linked the symptoms of IGD to symptoms of depression and anxiety among college students. **Methods:** The current study explored the relationships between self-reported symptoms of IGD, depression, and anxiety in two small, non-overlapping samples of college students, one collected before the pandemic (*n* = 52) and another during the global pandemic (*n* = 89). Data on the time spent gaming, IGD, depression, and anxiety symptoms were collected via anonymous online surveys at a small Mid-Western liberal arts university. The samples differed significantly in age, likely due to the smaller incoming first-year class size as a result of many families deciding to defer the start of college in 2020. **Conclusions:** These findings partially support past research suggesting a small to moderate association between self-reports of IGD and depression symptoms. While the pandemic does not appear to have greatly changed the overall number of self-reported symptoms experienced or the time spent playing video games between the two samples, it may have exacerbated the relationship between these variables within the sample. The stronger relationship between symptoms of depression or anxiety and the time spent playing video games in the later sample may be particularly concerning if the trend continues, as it may lead to additional problematic gaming behavior in the future.

## 1. Introduction

With an estimated 3.2 billion individuals playing video games worldwide [1] and the video game industry expected to continue growing [2], research interest in all aspects of gaming has increased. The literature has identified a broad array of effects of the video game experience on aggression [3], visuospatial attention [4], executive function [5], and more. While further research is needed to gain a complete picture of video game effects and the underlying mechanisms, it is well established that gaming can become problematic at least for some players [6,7]. Understanding the relationship between video game play and symptoms of mental health disorders is critically important. The current study examined the relationship between the time spent gaming and symptoms of internet gaming disorder (IGD), as well as symptoms of depression and anxiety. Due to the collection of samples pre- and post-COVID-19, differences in the relationships between these variables before and after the global pandemic were also examined.

### 1.1. Trends in Internet Gaming Disorder Prevalence

The prevalence of individuals experiencing symptoms of IGD has become a global concern. Clinical services have been established internationally, including in the United States with reSTART [https://www.restartlife.com/, accessed on 15 June 2023], the nation’s first treatment program for IGD. Moreover, a specialist clinic at the University Hospital of Geneva has had approximately 200 patients seek help for IGD since 2007 [8]. While the time spent gaming alone is not a perfect predictor of who will develop IGD, increased time spent online gaming (e.g., ten hours or more per day) can exacerbate the symptoms, with effects encompassing the social, academic, and vocational aspects of life.

In 2018, the World Health Organization (WHO) introduced a “gaming disorder” to the eleventh revision of the International Classification of Diseases (ICD-11) [9], which defines the diagnosis as a pattern of behavior severe enough to result in the critical impairment of the functioning of an individual’s personal, family, social, educational, occupational, or other essential life activities, as made evident for at least twelve months. Similarly, the Diagnostic and Statistical Manual of Mental Disorders (DSM-5-TR) [10] refers to the condition above as IGD, listing it in the section for conditions recommended for further research. Studies indicate that the prevalence of IGD varies, ranging from 0.7 to 27.5 percent, depending on the methodology used to measure it, with higher incidence rates observed among males compared to females [11]. The validity and reliability of survey instruments intended to measure IGD (based on the criteria outlined in the DSM-5) were tested in a Dutch meta-analysis in 2015 [12]. The results indicated that the dichotomous nine-item IGD scale yielded the most pragmatic results for diagnostic purposes, and it was therefore used in the current study to assess the prevalence of symptoms.

### 1.2. Symptoms of IGD, Anxiety, and Depression Among College Students

Mental health concerns among college students have been extensively documented, with a noticeable upward trend in the prevalence of symptoms [13]. A review spanning ten years revealed a significant increase in both the rate of diagnosis and treatment from 2007 (19%) to 2017 (24%) as well as a positive trend in students with lifetime diagnoses (22% to 35%) [14]. A study conducted in Belgium [15] found that approximately one-third of freshmen reported that mental health problems negatively impacted their academic performance. Recent work indicates that the pandemic has amplified mental health struggles in college students, with social isolation cited as a primary stressor [16,17]. In a U.S. study, 71% of students reported increased stress and anxiety due to the outbreak, with approximately 90% reporting negative impacts of the pandemic, such as difficulty concentrating, disruptions in sleep patterns, and decreased social interactions due to physical distancing measures [18].

Given the modest, but noticeable, increase in depression and anxiety among college students during the pandemic, it is possible that there may have been a similar increase in IGD. College students may have resorted to gaming as a coping mechanism for the complex reality of the pandemic, seeking social interaction in the virtual realm. Moreover, previous research has linked IGD to depression [19,20] and anxiety [21]. Teng and colleagues [22] examined the pandemic’s influence on the development of IGD as well as depressive and anxiety symptoms in a longitudinal study. Using two time intervals of T1 (before the pandemic; October–November 2019) and T2 (after the onset of the pandemic; April–May 2020) with a sample of over 1700 children and adolescents in Southwest China, it was revealed that children and adolescents both increased their videogame use at T1. Still, only adolescents exhibited a significantly increased IGD severity at T2. The authors interpreted their findings in support of the compensatory hypothesis, in which IGD is a result of psychological distress and decreased well-being in daily life. Their study also supports the interaction of person-affect-cognition-execution model (I-PACE), which views depressive and anxiety symptoms as predisposing variables that influence maladaptive gaming behavior.

### 1.3. The Current Study

The current study builds on the previous work examining the relationships between the time spent gaming, IGD, and depression and anxiety symptoms. Initially, a small sample of students self-reported their video game experience and symptoms in Fall of 2018 via an anonymous online survey. In Fall 2020, a unique opportunity arose to collect data on the same variables from a second sample of college students, allowing us to examine changes in the prevalence and correlations of mental health symptoms before and during the global pandemic. It should be noted, however, that the 2018 sample was not collected with the intention of conducting a longitudinal study and the 2020 sample was collected as part of a larger project on COVID-19 and mental health, without the initial intention to compare the two samples. We realized after the collection of the Fall 2020 sample that we had used several of the same measures, so our comparisons are largely exploratory, since neither sample was originally designed with a direct comparison in mind.

We focused on three central questions: (1) How prevalent were symptoms of IGD, depression, and anxiety in each sample? (2) What is the relationship between symptoms of the three conditions, and are the associations the same in each sample? (3) Is the amount of time spent playing video games weekly related to symptoms of depression and anxiety, and are these associations the same in each sample? Based on prior research, we hypothesized the following:

**H1:**  *The mean number of symptoms reported on the Beck depression inventory (BDI) and the generalized anxiety disorder 7-item scale (GAD-7) would be higher in the 2020 sample.*

**H2:**  *The internet gaming disorder scale (IGD), BDI, and GAD-7 scores would be positively correlated with one another and the correlations of IGD with BDI and GAD-7 would be larger in the 2020 sample.*

**H3:**  *The mean number of hours spent playing video games per week would be higher in the 2020 sample.*

**H4:**  *The mean number of hours spent playing video games per week would be positively correlated with the IGD, BDI, and GAD-7 scores and these correlations would be larger in the 2020 sample.*

## 2. Materials and Methods

### 2.1. Design and Participants

Data were collected through anonymous online surveys using Google Forms in two semesters: Fall 2018 and Fall 2020. The participants in both samples were recruited from a small Mid-Western university through Introduction to Psychology classes, announcements in university emails, and flyers on campus. The recruitment materials indicated the inclusion criteria (they must be 18 years of age or older and a student currently enrolled at the university), a brief description of the survey (they would be asked questions about their video game experience and symptoms of IGD, anxiety, and depression), and compensation (the opportunity to enter their email in a drawing for a USD 25 Amazon gift card). Recruitment via these notices and data collection lasted approximately two months (September to November) for both samples.

The Fall 2018 sample included fifty-three participants; the Fall 2020 sample included eighty-nine participants (demographic information about both samples is in Section 3.1). The university from which our sample was drawn had approximately 1400 students. Based on prior survey studies at this university, we anticipated that at least a quarter of the population would be exposed to the recruitment materials. We intended to collect a minimum of 100 responses to the surveys; this would reflect a 30% response rate, which we felt was reasonable given prior work with this population. Unfortunately, there were unanticipated challenges with recruitment and we fell short of that goal.

Learning from the experience in 2018, we increased the number of emails sent to campus organizations and expanded the posting of flyers into the student living spaces as well as academic buildings in 2020. The recruitment materials and Google Form also indicated that students who had completed similar surveys from our lab in the previous four years should not complete this survey. There were no further differences in the recruitment methods between the samples. The changes increased our yield in 2020, and while the anonymous nature of the Google Forms means that we cannot be sure there is no overlap in the samples, we have no evidence of significant overlap. The most significant source of participants in both samples was the Introduction to Psychology course, and therefore, it seems unlikely that there were many of the same students taking this course in Fall 2018 and Fall 2020. While we believe that our samples’ demographics reflect the population from which they were drawn (as described in the Results and explained in the Discussion), the findings should be interpreted with caution due to the small sample sizes.

### 2.2. Instruments

The materials were presented through an online Google Form maintained under the primary investigator’s university account. The participants read the consent form and checked a box to indicate agreement to participate in the study. Following consent, the participants answered demographic questions and completed each of the scales described in the paragraphs below. The participants had the option to skip any questions that they did not want to answer, and they could leave the study without submitting the form at any time. After the completion of all the questions and scales, the participants read a debriefing form that reminded them of the purpose of the study and asked them to confirm again their willingness to have their data included by clicking submit. If they did not want their data included, they could simply close the form without submitting. The measures reported in this paper are described below. Some additional measures were collected in the Fall 2020 sample (adult ADHD symptoms, COVID-19 fears, media multitasking habits), but as these were not collected in the first sample and are not relevant to the hypotheses of the current study, they will not be discussed further.

#### 2.2.1. Video Game Experience (VGE)

The participants reported their video gaming experience by answering eight questions (2018: α = 0.81; 2020: α = 0.89) about the number of hours spent playing video games during weekdays and weekends and twelve questions (2018: α = 0.82; 2020: α = 0.85) about their experience with video game genres (e.g., “Sports”, “Action/Adventure”, “Puzzle Games”) by stating “1: I never play it”, “2: I rarely play it”, “3: I occasionally play it”, “4: I sometimes play it”, “5: I often play it”, or “6: I always play it”. The total number of hours played per week was calculated [23].

#### 2.2.2. Internet Gaming Disorder Scale (IGD)

The participants answered “yes” or “no” to nine questions (2018: α = 0.67; 2020: α = 0.78) that screened for symptoms of IGD (e.g., “have there been periods when all you could think of was the moment that you could play a game?”). Responding yes to five or more symptoms is typically interpreted as consistent with a diagnosis of IGD [12].

#### 2.2.3. Beck Depression Inventory (BDI)

The participants reported their attitudes and symptoms of depression on a 20-item inventory (2018: α = 0.93; 2020: α = 0.92). The traditional BDI [24] has 21 items; the item asking about suicidal ideation was removed in the current study in compliance with the university’s Institutional Review Boards data collection policies. Previous work has questioned the usefulness of the suicidal ideation question when using the BDI [25,26] or other inventories [27] to measure depressive symptoms in a general population as opposed to a clinical sample. Each “item” consisted of four statements and the participants were instructed as follows: “Please read each group of statements carefully and then select the one statement in each group that best describes the way you have been feeling during the past two weeks, including today. If several statements in the group seem to apply equally well, select the highest number for that group”. Scores of 14–19 are in the mild mood disturbance range, 20–28 are moderate, and 29 and above are considered severe.

#### 2.2.4. Generalized Anxiety Disorder 7-Item Scale (GAD-7)

The participants reported how often they had been bothered by seven (2018: α = 0.89; 2020: α = 0.92) problems (e.g., “trouble relaxing”, “feeling nervous, anxious, or on edge”) over the last two weeks using a scale from 0 (not at all sure) to 3 (nearly every day). There was also an eighth question that asked how difficult the problems have made work, home life, or interpersonal relationships on a scale of not difficult at all to extremely difficult. Cutoff scores of five, ten, and fifteen correspond to mild, moderate, and severe anxiety, respectively [28].

### 2.3. Data Analysis

The means and standard deviations were calculated for all measures separately for each sample and the means were compared with Student’s *t*-test and Cohen’s *d*. Cohen’s *d* is an effect size measure and can be used to quantify the size of the difference between two groups for a variable. By dividing the difference between the means of the two groups by the common standard deviation, the effect size between different samples and studies can be compared. Cohen’s *d* values of 0.2, 0.5, and 0.8 have been established as conventions for classifying effect sizes as small, medium, and large [29]. Pearson *r* correlations were calculated between variables of interest separately by sample, and then the samples were compared using Fisher’s *r* to *z* transformation [30]. For some analyses of the IGD, BDI, and GAD-7, the continuous scores were converted to categorical variables and comparisons between samples were made with the chi-square test of independence. SPSS 29 and Microsoft Office Excel 2021 were used to complete the analyses. A significance level of 0.05 was used for all analyses.

### 2.4. Ethical Considerations

All the participants gave their informed consent via the online survey by checking a box to indicate that they had read and understood the information provided and agreed to participate in the study. If a participant did not check this box, then the Google Form did not progress to the rest of the survey. The study was conducted in accordance with the Declaration of Helsinki, and the protocol was approved by the Institutional Review Board of Ohio Wesleyan University (1810.010, 2010.017). For their time, the participants who chose to do so provided their email in a separate form to be entered into a lottery drawing for one of four Amazon gift cards with a value of USD 25.

## 3. Results

### 3.1. Demographic Comparisons Between Samples

The Fall 2018 sample included fifty-three participants. The data from one participant were excluded due to technical issues. The mean age of the participants was 18.75 years (*SD* = 1.01) and they predominantly identified as female (37 female, 12 male, 1 other, 2 unknown).

The Fall 2020 sample included eighty-nine participants. The data from one participant were excluded due to technical issues. The mean age of the participants was 20.66 years (*SD* = 1.73) and they predominantly identified as female (64 female, 20 male, 3 transgender, 1 unknown).

The age of the participants in the 2018 (M = 18.75 years, SD = 1.01) and 2020 (M = 20.66 years, SD = 1.73) studies differed significantly; *t*(138) = 7.25, *p* < 0.001. The effect size, as measured by Cohen’s *d*, was *d* = 1.29, indicating a large effect. In Fall 2020, the number of first-year students entering the university was smaller than typical (approximately 300 students compared to over 400 students in prior and subsequent years) due to the pandemic. This likely impacted the mean age of the population from which our sample was drawn.

The proportion of female students in the 2018 (71.2%) and 2020 (72.7%) samples did not differ significantly; *t*(138) = 0.01, *p* = 0.92. The effect size was *d* = 0.02, indicating a very small effect. The student population from which our sample was drawn was skewed slightly towards females (60%), and the population of psychology students at this institution was skewed even more towards females (83%). Given that Introduction to Psychology and other upper-level courses were targeted as part of the recruitment strategy, it is unsurprising that our samples were predominantly female.

### 3.2. Prevalence of Symptoms

The mean number of symptoms of IGD was relatively low (Table 1) and did not differ significantly between the samples; *t*(138) = 1.57, *p* = 0.12. The effect size was *d* = 0.28, indicating a small effect. Across both samples, 12% of the participants reported experiencing five or more of the symptoms, which is consistent with the prevalence of IGD reported in past research [6,11]. The proportion of the participants who reported five or more symptoms (Table 2) did not differ by sample; *X*^2^ (1, *N* = 140) = 1.54, *p* = 0.22. Although the raw means suggested an increase in prevalence, the statistical analysis yielded no indication that COVID-19 significantly increased the prevalence of IGD symptoms.

The mean number of symptoms reported on the BDI was in the mild mood disturbance range (Table 1) and did not differ significantly between the samples; *t*(138) = 1.48, *p* = 0.14. The effect size was *d* = 0.26, indicating a small effect. The proportion of participants meeting the criteria for mild, moderate, or severe depression (Table 2) also did not differ by sample; *X*^2^ (3, *N* = 140) = 2.24, *p* = 0.53. These findings are inconsistent with H1; it does not appear that the mean number of symptoms or the proportion of the sample meeting the criteria for the cut-offs was significantly greater in 2020 compared to 2018.

The mean number of symptoms reported on the GAD-7 was in the moderate anxiety range (Table 1) and the means did not significantly differ between the samples; *t*(138) = 1.04, *p* = 0.30. The effect size was *d* = 0.18, indicating a very small effect. The proportion of participants meeting the criteria for mild, moderate, or severe anxiety (Table 2) did not differ by sample; *X*^2^ (3, *N* = 140) = 1.57, *p* = 0.67. Similar to the BDI, these findings are inconsistent with H1; it does not appear that the prevalence of anxiety symptoms or the proportion of the sample meeting the criteria for the cut-offs was significantly greater in 2020 than 2018.

### 3.3. Relationship Between Symptoms of Anxiety, Depression, and Internet Gaming Disorder

The correlations between the scales are reported separately for Fall 2018 and 2020 (Table 3). To compare the correlations between the samples, the *r* values were transformed into *z*-scores and compared to the critical value, with the significance level set to 0.05.

There was a weak correlation between the IGD and BDI scores in both samples (Table 3). This association was not significantly different between the samples; *z*_(observed)_ = 0.12, *p* = 0.90. The correlation between the IGD and GAD-7 scores was not statistically significant in the samples, nor did the relationship differ between samples; *z*_(observed)_ = −0.06, *p* = 0.95. Together, these findings partially support H2, as IGD and the BDI were significantly correlated. However, the IGD and GAD-7 scores were not correlated and there were no differences between the samples for IGD correlations with either the BDI or the GAD-7.

As expected, there was a strong correlation between the BDI and GAD-7 scores in both samples (Table 3), supporting H2. However, the relationship between depression and anxiety was not significantly different between the samples (*z*_(observed)_ = −0.74, *p* = 0.46), which is inconsistent with H2.

The pattern of results here is both consistent and inconsistent with H2 and previous work [19,20,21]. The close association between the symptoms of depression and anxiety was replicated, as well as a weak positive association between the symptoms of IGD and depression. However, there was no significant relationship between the symptoms of IGD and anxiety, and the strength of the relationships did not appear to be impacted by the pandemic.

### 3.4. Relationship Between Hours per Week and Symptoms

The average hours per week reported was not significantly different in 2018 (*M* = 13, *SD* = 13.16) and 2020 (*M* = 14.98, *SD* = 19.89); *t*(138) = 0.64, *p* = 0.52. This is inconsistent with H3. The effect size was *d* = 0.11, indicating a very small effect. Partially consistent with H4, there was a moderate correlation between the hours per week and IGD (Table 3) in both samples, with no significant difference between the samples; *z*_(observed)_ = −0.81, *p* = 0.42.

The correlation (Table 3) between the hours per week and the BDI was not significant in the 2018 sample. However, there was a significant positive correlation in the 2020 sample; *z*_(observed)_ = −2.53, *p* = 0.01. Similarly, the correlation between the hours per week and the GAD-7 was not significant in the 2018 sample, but there was a significant positive correlation in the 2020 sample; *z*_(observed)_ = −2.27, *p* = 0.02. These findings are partially consistent with H4.

The data suggest that the amount of time spent playing video games weekly has a positive correlation with IGD symptoms, but this relationship did not appear to be impacted by the pandemic, partially supporting our hypotheses. Interestingly, the relationships between amount of time playing and depression and anxiety were significantly stronger during the pandemic compared to before. Given that there was not an overall increase in the time spent playing or the prevalence of symptoms reported on the BDI or GAD-7 between the samples, the change in the strength of the relationships may have been driven by other factors, such as using video games to escape. Specifically, increased video game play combined with maladaptive coping strategies may indicate a higher risk of developing IGD, suggesting that coping plays a crucial role in the relationship between IGD, depression, stress, and anxiety, but does not fully account for the relationship [31]. Unfortunately, neither sample was asked to report their reasoning for gaming, so the current dataset cannot directly answer that question.

## 4. Discussion

The current study tested four hypotheses regarding the prevalence and relationships among self-reported symptoms of IGD, depression, and anxiety in two samples before and during the COVID-19 pandemic. The findings partially support our hypotheses and past research. While the mean number of symptoms reported on the IGD, BDI, and GAD-7 were not significantly higher in the 2020 sample (H1), there was a small positive correlation between the reported symptoms on the IGD and BDI (H2). Likewise, our data found no significant increase in the hours spent playing video games per week (H3), but there were changes in the relationship between the time spent playing and other measures (H4). We discuss the findings, implications, and limitations of this study in more detail in the following paragraphs.

The number of symptoms reported by our samples was consistent with mild to moderate levels of depression and anxiety, with no significant differences between the Fall 2018 and Fall 2020 samples. While the lack of change is inconsistent with the work conducted on much larger samples of college students [14,15,16,17,18], the overall prevalence of the symptoms is consistent. College students experience alarming levels of depression and anxiety symptoms. Similarly, the IGD symptoms did not increase from the 2018 to 2020 samples, but the overall prevalence, with around 12% reporting five or more symptoms, is consistent with other findings [6,11]. As others have suggested [32], the mental wellness of college students needs to be a high priority for colleges and universities. Various political, environmental, and economic factors may exacerbate mental health symptoms in an already vulnerable population, placing the faculty, staff, and administration on college campuses on the frontline of a mental health crisis.

Unexpectedly, there was only a small correlation between symptoms of IGD and depression, and a nonsignificant correlation between symptoms of IGD and anxiety. This was surprising because other studies have found stronger associations between these disorders [19,20,21]. A powerful longitudinal study conducted by Teng and colleagues [22] suggests that the relationship among these disorders is strong and important for understanding the development of IGD. Their work supports the idea that IGD is a result of psychological distress and decreased well-being in daily life, as expected in the compensatory hypothesis and the I-PACE model of depressive and anxiety symptoms as predisposing variables for IGD. The current study did not replicate as strong of an association between the symptoms of IGD, depression, and anxiety. Our sample sizes were considerably smaller and more female-identifying than those of past research and the current study did not target clinical populations for any of the disorders, factors that likely contributed to the smaller correlations. There are benefits to studying these variables in both clinical and non-clinical samples, and in fact, a comparison of those two might be a fruitful avenue for future research. Perhaps not surprisingly, given the small and nonsignificant relationships, there were also no significant changes before and during the pandemic. It appears that the prevalence and relationships among the symptoms of these disorders were stable in this small population of Mid-Western and predominantly female college students.

While the relationships with IGD were not quite as expected, examining the amount of time spent playing video games yielded results more consistent with those of Teng and colleagues [22], the compensatory hypothesis, and the I-PACE model. While there were no overall increases in the amount of time spent playing video games between the 2018 and 2020 samples, there were significant changes in the relationship between gaming and symptoms of depression and anxiety. Both depression and anxiety symptoms were positively correlated with the time spent gaming during the pandemic. While we found no increase in the prevalence of symptoms in our samples during the pandemic, it is possible that social isolation led students to turn to gaming for social interaction and/or to escape the difficult realities of the pandemic. Using gaming for these reasons would be consistent with the compensatory hypothesis and the I-PACE model. It will be important for future research to include measures of gaming motivations, well-being, and psychological distress to increase our understanding of how and why gaming becomes problematic. This study may have captured a pre-IGD state, in which the link between the time spent gaming and mental health symptoms is growing stronger. If the pattern continues, it could lead to the development of IGD. We argue that researchers and clinicians should consider a closer examination of a pre-IGD state. There may well be behavioral or physiological markers that indicate when an individual is developing problematic gaming behaviors, before it is diagnosable. Identifying an individual in this state could lead to more successful interventions.

The current study has important limitations to keep in mind. The sample size was small compared to many of the other studies conducted on this topic, so there may have been a limited power to detect some of the relationships [6,11,14,15,16,17,18,19,20,21]. The sample was also predominantly female, and past work suggests that IGD is more prevalent among males than females [6,11]. The overall prevalence of IGD found here fits with other work, but it is very possible that the relationships among symptoms vary by sex. The Fall 2020 sample was measured early in the pandemic, so it is possible that some of the increases in the symptoms and relationships among disorders were not yet evident. This seems more likely, given that the relationships between the time spent gaming and symptoms of depression and anxiety were clearly becoming stronger even though there was no measurable difference in IGD symptoms. Finally, because we did not originally set out in 2018 to measure the impact of a global pandemic, we did not include all the measures needed to understand the patterns that emerged. For example, we did not collect data on the motivations for playing video games, which may have been especially useful for testing the compensatory hypothesis and the I-PACE model given what we now know about the increase in social isolation during the early days of COVID-19 [22]. Furthermore, because of the unanticipated pandemic, the 2018 sample set was not intended to be used for longitudinal purposes, and therefore, we had to collect an independent sample in 2020. Thus, this may partially explain the weaker correlations. While we are confident that our samples are representative of the specific, small, Mid-Western population of college students from which they originated, there are very likely important distinctions between this population and a broader population, or even a broader population of college students in the United States specifically. Larger-scale online studies might better capture a nationally or globally representative sample.

## 5. Conclusions

In conclusion, while the pandemic did not appear to greatly change the number of symptoms experienced or the time spent playing video games in our samples, it may have exacerbated the relationship between these variables. The stronger relationship between depression, anxiety, and the time spent playing video games may be particularly concerning considering the compensatory hypothesis and the I-PACE model [22]. It would be worthwhile for researchers and clinicians to consider how large-scale events might influence the relationships between video game playing and mental wellness in research and in practice. The findings of this study should be interpreted cautiously due to limitations in the design and the small sample size, but we hope that future work can address some of these limitations.

## Figures and Tables

**Table 1:** Mean number of symptoms reported for IGD, BDI, and GAD-7.

Scale	2018	2020
IGD	1.71 (*SD* = 1.62)	2.24 (*SD* = 2.07)
BDI	13.52 (*SD* = 10.26)	16.30 (*SD* = 10.96)
GAD-7	9.63 (*SD* = 5.37)	10.68 (*SD* = 5.93)

**Table 2:** Percentage of sample at cutoffs for IGD, BDI, and GAD-7.

Scale	2018	2020
IGD	7.7%	14.8%
BDI	14% (mild); 15% (mod); 12% (severe)	17% (mild); 21% (mod); 16% (severe)
GAD-7	25% (mild); 33% (mod); 21% (severe)	25% (mild); 25% (mod); 30% (severe)

**Table 3:** Correlations between reported symptoms of IGD, BDI, and GAD-7 and hours per week.

Scales	2018 *r, p*	2020 *r, p*
IGD × BDI	0.267, 0.056	0.247, 0.020
IGD × GAD-7	0.122, 0.387	0.130, 0.228
BDI × GAD-7	0.707, <0.001	0.766, <0.001
hours per week × IGD	0.444, <0.001	0.555, <0.001
hours per week × BDI	−0.031, 0.827	0.403, <0.001
hours per week × GAD-7	−0.132, 0.351	0.265, 0.013

## Data Availability

The data presented in this study are available from the corresponding author upon request. The data are not publicly available due to university policy.

## References

[1] wepc.com. https://www.wepc.com/.

[2] Next Generation Technologies. https://www.ngtnet.net/.

[3] Burkhardt J., Lenhard W. (2022). A meta-analysis on the longitudinal, age-dependent effects of violent video games on aggression. Media Psychol..

[4] Matern M.F., van der Westhuizen A., Mostert S.N. (2020). The effects of video gaming on visual selective attention. S. Afr. J. Psychol..

[5] Sampalo M., Lázaro E., Luna P.-M. (2023). Action video gaming and attention in young adults: A systematic review. J. Atten. Disord..

[6] Liao Z., Huang Q., Huang S., Tan L., Shao T., Fang T., Chen X., Lin S., Qi J., Cai Y. (2020). Prevalence of internet gaming disorder and its association with personality traits and gaming characteristics among Chinese adolescent gamers. Front. Psychiatry.

[7] Smirni D., Garufo E., Di Falco L., Lavanco G. (2021). The playing brain. The impact of video games on cognition and behavior in pediatric age at the time of lockdown: A systematic review. Pediatr. Rep..

[8] Saunders J.B., Hao W., Long J., King D.L., Mann K., Fauth-Bühler M., Rumpf H.-J., Bowden-Jones H., Rahimi-Movaghar A., Chung T. (2017). Gaming disorder: Its delineation as an important condition for diagnosis, management, and prevention. J. Behav. Addict..

[9] International Classification of Diseases, Eleventh Revision (ICD-11), World Health Organization (WHO). 2019/2021. https://icd.who.int/browse11.

[10] American Psychiatric Association (2013). Diagnostic and Statistical Manual of Mental Disorders.

[11] Mihara S., Higuchi S. (2017). Cross-sectional and longitudinal epidemiological studies of internet gaming disorder: A systematic review of the literature. Psychiatry Clin. Neurosci..

[12] Lemmens J.S., Valkenburg P.M., Gentile D.A. (2015). The internet gaming disorder scale. Psychol. Assess..

[13] Buizza C., Bazzoli L., Ghilardi A. (2022). Changes in college students mental health and lifestyle during the COVID-19 pandemic: A systematic review of longitudinal studies. Adolesc. Res. Rev..

[14] Sarah Lipson S.L., Emily Lattie E.L., Daniel Eisenberg D.E. (2019). Increased rates of mental health service utilization by U.S. college students: 10-year population-level trends (2007–2017). Psychiatr. Serv..

[15] Bruffaerts R., Mortier P., Kiekens G., Auerbach R.P., Cuijpers P., Demyttenaere K., Green J.G., Nock M.K., Kessler R.C. (2018). Mental health problems in college freshmen: Prevalence and academic functioning. J. Affect. Disord..

[16] Adams K., Saunders K., Keown-Stoneman C., Duffy A. (2021). Mental health trajectories in undergraduate students over the first year of university: A longitudinal cohort study. BMJ.

[17] McLafferty M., Brown N., McHugh R., Ward C., Stevenson A., McBride L., Brady J., Bjourson A., O’Neil S., Walsh C. (2021). Depression, anxiety and suicidal behaviour among college students: Comparisons pre-COVID-19 and during the pandemic. Psychiatry Res. Comm..

[18] Cao W., Fang Z., Hou G., Han M., Xu X., Dong J., Zheng J. (2020). The psychological impact of the COVID-19 epidemic on college students in China. Psychiatry Res..

[19] Ostinelli E.G., Zangani C., Giordano B., Maestri D., Gambini O., D’agostino A., Furukawa T.A., Purgato M. (2021). Depressive symptoms and depression in individuals with internet gaming disorder: A systematic review and meta-analysis. J. Affect. Disord..

[20] Ryu H., Lee J.-Y., Choi A., Park S., Kim D.-J., Choi J.-S. (2018). The relationship between impulsivity and internet gaming disorder in young adults: Mediating effects of interpersonal relationships and depression. Int. J. Environ. Res. Public Health.

[21] Fazeli S., Zeidi I.M., Lin C.-Y., Namdar P., Griffiths M.D., Ahorsu D.K., Pakpour A.H. (2020). Depression, anxiety, and stress mediate the associations between internet gaming disorder, insomnia, and quality of life during the COVID-19 outbreak. Addict. Behav. Rep..

[22] Teng Z., Pontes H.M., Nie Q., Griffiths M.D., Guo C. (2021). Depression and anxiety symptoms associated with internet gaming disorder before and during the COVID-19 pandemic: A longitudinal study. J. Behav. Addict..

[23] Bailey K., West R. (2017). Did I do that? The association between action video gaming experience and feedback processing in gambling task. Comput. Hum. Behav..

[24] Beck A.T., Ward C.H., Mendelson M., Mock J., Erbaugh J. (1961). An inventory for measuring depression. Arch. Gen. Psychiatry.

[25] Poole H., Bramwell R., Murphy P. (2009). The utility of the Beck Depression Inventory Fast Screen (BDI-FS) in a pain clinic population. Eur. J. Pain.

[26] Golden J., Conroy R.M., O’Dwyer A.M. (2007). Reliability and validity of the hospital anxiety and depression scale and the beck depression inventory (full and fast screen scales) in detecting depression in persons with hepatitis C. J. Affect. Disord..

[27] Gómez-Gómez I., Benítez I., Bellón J., Moreno-Peral P., Oliván-Blázquez B., Clavería A., Zabaleta-Del-Olmo E., Llobera J., Serrano-Ripoll M.J., Tamayo-Morales O. (2022). Utility of PHQ-2, PHQ-8 and PHQ-9 for detecting major depression in primary health care: A validation study in Spain. Psychol. Med..

[28] Spitzer R.L., Kroenke K., Williams J.B.W., Lowe B. (2006). A brief measure for assessing generalized anxiety disorder. Arch. Intern. Med..

[29] Cohen J., Hillsdale N.J. (1988). Statistical Power Analysis for the Behavioral Sciences.

[30] Mendoza J.L. (1993). Fisher transformations for correlations corrected for selection and missing data. Psychometrika.

[31] Loton D., Borkoles E., Lubman D., Polman R. (2015). Video game addiction, engagement, and symptoms of stress, depression, and anxiety: The mediating role of coping. Int. J. Ment. Health Addict..

[32] Cerutti R., Spensieri V., Amendola S., Biuso G.S., Renzi A., Gambardella A., Tambelli R. (2023). Clinical symptoms in pre-COVID-19 pandemic versus COVID-19 pandemic samples of Italian university students. Minerva Psychiatry.

